# Nutritionally Derived Metabolic Cues Typical of the Obese Microenvironment Increase Cholesterol Efflux Capacity of Adipose Tissue Macrophages

**DOI:** 10.1002/mnfr.201800713

**Published:** 2018-11-20

**Authors:** Marcella E. O'Reilly, Sarina Kajani, Jessica C. Ralston, Yvonne M. Lenighan, Helen M. Roche, Fiona C. McGillicuddy

**Affiliations:** ^1^ Nutrigenomics Research Group School of Public Health Physiotherapy and Sports Science University College Dublin Dublin 4 Ireland; ^2^ UCD Institute of Food and Health University College Dublin Dublin 4 Ireland; ^3^ Diabetes Complications Research Centre UCD Conway Institute and School of Medicine University College Dublin Dublin 4 Ireland

**Keywords:** ABCA1, adipose tissue macrophage (ATM), cholesterol efflux capacity (CEC), glucose, M1 polarization

## Abstract

**Background:**

Cholesterol retention within plasma membranes of macrophages is associated with increased inflammatory signaling. Cholesterol efflux via the transporters ABCA1, ABCG1, and SR‐BI to high‐density lipoprotein (HDL) particles is a critical mechanism to maintain cellular cholesterol homeostasis. Little is known about the impact of the obese microenvironment on cholesterol efflux capacity (CEC) of macrophages. In this study, the CEC of obese‐derived primary adipose‐tissue macrophages (ATM) is evaluated and the in vivo microenvironment is modeled in vitro to determine mechanisms underlying modulated CEC.

**Materials and Methods:**

F4/80^+^ ATM are labeled with ^3^H‐cholesterol ex vivo, and CEC and ABCA1/ABCG1 protein levels are determined. Total, ABCA1‐dependent, and ABCA1‐independent CECs are determined in J774 macrophages polarized to M1 (LPS&IFNγ), M2 (IL‐4&IL‐13), or metabolic phenotypes (glucose, insulin, and palmitic acid).

**Results:**

Obese ATM exhibit enhanced CEC and ABCA1 and ABCG1 expression compared to lean ATM. In contrast, ABCA1‐CEC is suppressed from M1 polarized macrophages compared to untreated in vitro, by activation of the JAK/STAT pathway. Incubation of macrophages in vitro in high glucose augments cAMP‐induced ABCA1 protein expression and ABCA1‐CEC.

**Conclusions:**

These novel findings demonstrate remarkable plasticity of macrophages to respond to their environment with specific modulation of ABCA1 depending on whether classical pro‐inflammatory or metabolic cues predominate.

## 1 Introduction

Recruitment of pro‐inflammatory macrophages is evident in obese adipose tissue^1^ and is associated with a state of chronic low‐grade inflammation and insulin resistance.^2^ Conditional ablation of CD11C^+^ cells after onset of obesity in turn normalizes insulin sensitivity.^3^ Cholesterol acquisition within macrophages is a classic hallmark of cardiometabolic disease and the main purported mechanism to reduce lipid burden is via cholesterol efflux onto high‐density lipoprotein (HDL) particles.^4^ Lipid‐laden macrophages efflux cholesterol onto lipid‐poor ApoA1 and small HDL particles via ABCA1 or onto larger HDL particles via scavenger receptor‐BI (SR‐BI) and ABCG1.^4^ Reduction in cellular cholesterol efflux via ABCA1 and/or ABCG1 enhances pro‐inflammatory Toll like receptor (TLR) signaling due to cholesterol enrichment within the plasma membrane.^5^, ^6^

Little is understood about the impact of the obese microenvironment on cholesterol efflux capacity (CEC) of macrophages in vivo. We previously demonstrated that obese‐derived adipose stromal vascular cells, which contain the immune cell populations, exhibit a heightened inflammatory response to lipopolysaccharide ex vivo compared to lean‐derived stromal vascular cells.^7^ Furthermore, a phenotypic switch in adipose tissue macrophages (ATM) is observed in obesity with increased numbers of pro‐inflammatory M1 macrophages evident relative to pro‐resolving/anti‐inflammatory M2 macrophages.^1^ We originally hypothesized that CEC would be suppressed from obese ATM resulting in cellular cholesterol retention within the plasma membrane and sustained pro‐inflammatory signaling. We further hypothesised that reduced CEC would be more pronounced in M1 macrophages compared to M2 macrophages and evaluated this in vitro due to limitations in primary cell numbers. In contrast to our hypothesis, we found CEC was significantly enhanced in obese‐ATM compared to lean‐ATM but was markedly suppressed in classically polarized M1 macrophages in vitro. Incubation of macrophages with metabolic stressors (glucose, insulin, and palmitic acid), that are augmented in the obese microenvironment, could re‐capitulate the findings from primary obese ATM and suggest that microenvironmental factors are governing ABCA1/ABCG1 protein expression within the obese state. Increased cholesterol efflux from obese‐ATM may be a protective mechanism to both protect against lipotoxicity, due to acquisition of lipids within the local microenvironment, as well as lower inflammation by depleting membrane cholesterol content.

## 2 Experimental Section

### 2.1 Materials

Cholesterol [1,2‐^3^H(N)] was purchased from Perkin–Elmer Analytical Sciences (Ireland). Cell culture material was purchased from Lonza (Slough, UK). Diets were purchased from Research Diets Incorporated (USA). JAK inhibitor (catalog no.: 420099) was purchased from Calbiochem (USA). CREB inhibitor (666‐15 catalog no.: 538341) was purchased from Merck (Germany). Polarizing cytokines (IFN‐γ, IL‐4, IL‐13, IL‐1β, IL‐10, IL‐6, TNF‐α, and Leptin) were purchased from R&D bioscience (USA). LPS (catalog no.: ALX‐581‐012‐L002) was purchased from ENZO Biochem Inc. (USA). All other reagents, unless otherwise stated, were from Sigma–Aldrich Ltd. (Ireland).

### 2.2 Animals

Male C57BL/6j mice (Harlan, UK) fed an HFD (60% kCal from fat, catalog no.: D12492, Research Diets), or a micronutrient‐matched low‐fat diet (LFD) (10% kCal from fat, catalog no.: D12450B, Research Diets) for 12 weeks were anesthetized and euthanized by cervical dislocation. Ethical approval was obtained from UCD Animal Research Ethics Committee, number P12‐09 and mice were maintained according to Directive 2010/63/EU and Irish Statutory Instrument 543 of 2012.

### 2.3 Cell Culture

J774.2 murine macrophages were maintained in DMEM (Lonza catalog no.: BE12‐604F) with 10% Fetal bovine serum (FBS) and 1% penicillin/streptomycin.

### 2.4 Cholesterol Efflux Assay

J774 macrophages were labeled for 24 h with ^3^H‐cholesterol (1μCi mL^–1^) in RPMI media (11 mm glucose). Cells were equilibrated for 18 h in DMEM or d‐glucose‐free DMEM (Gibco, catalog no.: 11965025) containing 0.2% BSA ± cAMP (0.3 mm) ± polarizing cytokines. J774 macrophages did not express ABCA1 basally—cAMP was used to induce ABCA1 specifically. The difference in efflux from cells stimulated in the presence or absence of cAMP represented ABCA1‐dependent efflux. ABCA1‐independent efflux was derived from untreated (‐cAMP) cells. Total efflux was calculated from cells treated + cAMP and was a measure of efflux via all pathways. Percentage ^3^H‐cholesterol efflux to FBS (2.5%) was monitored over 4 h by liquid scintillation counting. Percentage efflux to minimal essential media was determined and subtracted as background control.

### 2.5 Macrophage Polarization

Macrophages were polarized for 18 h to M1 phenotype using LPS (100/10 ng mL^–1^) + IFNγ (20/2 ng mL^–1^), to M2 phenotype using IL‐4 (20 ng mL^–1^) + IL‐13 (20 ng mL^–1^) in DMEM containing 25 mm d‐glucose (Lonza, Cat.#BE12‐604F) or to MMe using insulin (1 nm), glucose (4/25 mm) + palmitic acid (1/5 μm) in d‐glucose free DMEM (Gibco, catalog no.: 11966025). For pathway analysis studies, cells were pretreated with JAK Inhibitor (0.1–5 μm) (Calbiochem catalog no.: 420099) or LXR agonist T0901317 (10 μm) (Sigma catalog no.: 2320) prior to cotreatment with M1 polarizing cytokines. Cells were pretreated with CREB inhibitor (5 μm) (666‐15, Tocris Bioscience catalog no.: 5661) for 42 h prior to incubation ± cAMP (0.3 mm) in high (25 mm) and glucose‐free conditions.

### 2.6 F4/80^+^ Cell Isolation

Epididymal adipose tissue fat pads were minced and stromal Vascular Fraction (SVF) separated by collagenase (2 mg mL^–1^) digestion. SVF cells were incubated with a Fluorescein isothiocyanate (FITC)‐conjugated F4/80 primary antibody (BioRad catalog no.: MCA497F) at 4 °C for 30 min. Cells were washed, centrifuged, and incubated with MACS anti‐FITC microbeads (Miltenyibiotec catalog no.: 130‐048‐701) for a further 15 min and were separated using a MACS LS column according to manufacturer's instructions. F4/80^+^ cells (7 × 10^5^ cells per well) were incubated with ^3^H‐cholesterol (1μCi mL^–1^) overnight and equilibrated in 0.2% BSA containing media for a further 18h. Percentage ^3^H‐cholesterol efflux to ApoA1 (20 μg mL^–1^; an indirect measure of ABCA1‐dependent efflux) and serum (2.5%; a measure of total efflux via all transporters) over 4 h was determined. Cellular protein was harvested in RIPA buffer for immunoblot analysis.

### 2.7 Immunoblot Analysis

Cellular protein was harvested in RIPA buffer (Millipore) containing complete mini protease inhibitors (Roche Ltd, Ireland). Protein concentration was quantified by Pierce BCA assay (Bio‐Rad Laboratories Inc., USA). Equal concentrations of lysate (10 μg) were separated by SDS‐PAGE, transferred to nitrocellulose membranes, blocked, and incubated overnight (at 4 °C) in primary antibody.^7^ Blots were probed with antibodies to ABCA1, ABCG1, SR‐BI (Novus Biologicals, UK, catalog no.:, NB400‐15, NBP2‐54682m, and NB400‐105, respectively), phosphorylated (P)‐STAT1, whole‐cell (WC)‐STAT1, P‐STAT3, WC‐STAT3, GAPDH, or β‐actin (Cell Signalling, MA, USA, catalog no.: 8826S, 14994S, 9145S, 12640S, 5174S, and 3700P, respectively). Blots were washed, incubated in secondary antibody, and visualized using Pierce ECL Western Blotting Substrate (Thermo Fisher Scientific Inc., USA). For densitometry analysis, bands were quantitated using Image J software and normalized to either GAPDH, β‐actin, or Ponceau at 70 kDa as indicated. Densitometry data are presented in Figures S3–S7, Supporting Information.

### 2.8 Gene Expression Analysis

RNA was extracted from cells using TRI‐Reagent. Single‐stranded cDNA was prepared using High‐Capacity cDNA Archive Kit (Applied Biosystems, UK). Labeled primers and probes and TaqMan Universal Mastermix were obtained from Applied Biosystems. mRNA expression was quantified by real‐time PCR (RT‐PCR) on an ABI 7700 Sequence Detection System (Perkin–Elmer Applied Biosystems). To control for between‐sample variability, mRNA levels were normalized to *Gapdh* for each sample by subtracting the Ct for *Gapdh* from the Ct for the gene of interest producing a ΔCt value. The ΔCt for each treatment sample was compared to the mean ΔCt for control samples using the relative quantification 2‐(ΔΔCt) method to determine fold‐change.

### 2.9 Statistical Analysis

Data were presented as mean ± SEM and represented three independent experiments performed in triplicate. Data were tested for normal Gaussian distribution by Shapiro–Wilk test. Normally distributed data were tested by one‐way or two‐way analysis of variance (ANOVA) as appropriate and when significant Bonferroni post hoc tests were applied. Data‐sets with nonnormally distributed data underwent Kruskal–Wallis testing with Dunn's post hoc test. For comparison of data between two groups, an unpaired *t*‐test was applied to normally distributed data while a Mann–Whitney *U* test was applied to nonnormally distributed data. GraphPad Prism 5 (GraphPad Software Inc., CA) was used for statistical analysis.

## 3 Results

### 3.1 Obese‐Derived ATM Exhibits Increased CEC and Cholesterol Transporter Expression Compared to Lean‐ATM

**Figure 1:**
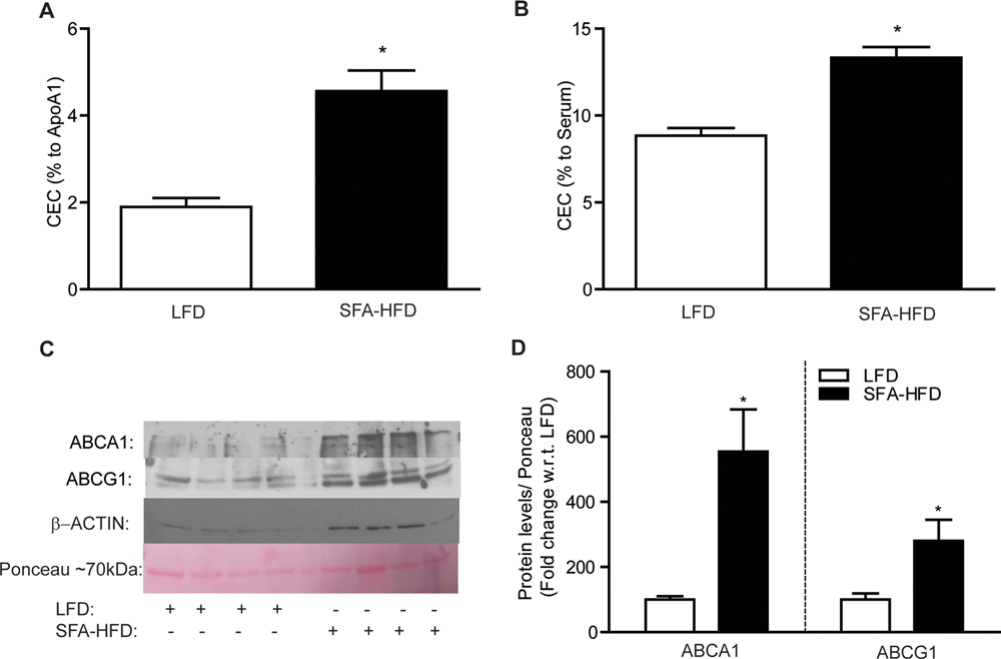
Primary ATM exhibit enhanced CEC. Adipose‐derived F4/80^+^ cells were isolated from C57BL/6j mice after 12 weeks on HFD or LFD. F4/80^+^ cells were labeled with ^3^H‐cholesterol (1 μCi mL^–1^) in RPMI media for 24 h then equilibrated in 0.2% BSA for 24 h. Cholesterol efflux to A) ApoA1 (25 μg mL^–1^) and B) serum (2.5%) was determined over 4 h (*p < 0.05 w.r.t. LFD). C) Protein expression of ABCA1 and ABCG1 and ponceau loading control in F480^+^ cells was determined by immunoblot analysis and D) corresponding densitometry is presented (*p < 0.05 w.r.t. LFD, *n* = 4).

Macrophages infiltrating obese adipose tissue are predominantly of M1 phenotype^1^ but are exposed to a unique metabolic microenvironment compared to circulating cells.^8^ We therefore sought to investigate the efflux capacity of primary F4/80^+^ macrophages from obese versus lean adipose tissue. F4/80^+^ cells from obese mice exhibited significantly increased CEC to ApoA1 and serum compared to F4/80^+^ cells from lean mice (**Figure** 1A and B), coincident with increased ABCA1 and ABCG1 protein levels (Figure 1C and D). The obese microenvironment is enriched for pro‐inflammatory factors including TNF‐α, IL‐1β, and IL‐6 and metabolic stressors such as palmitic acid, glucose, and insulin. We therefore took a reductionist approach in vitro to determine potential mechanisms for the enhanced CEC observed in vivo by determining the effects of macrophage polarization, local inflammatory cytokines, and metabolic stressors on macrophage CEC.

### 3.2 Classically Activated M1 Macrophages Exhibit Reduced CEC

**Figure 2:**
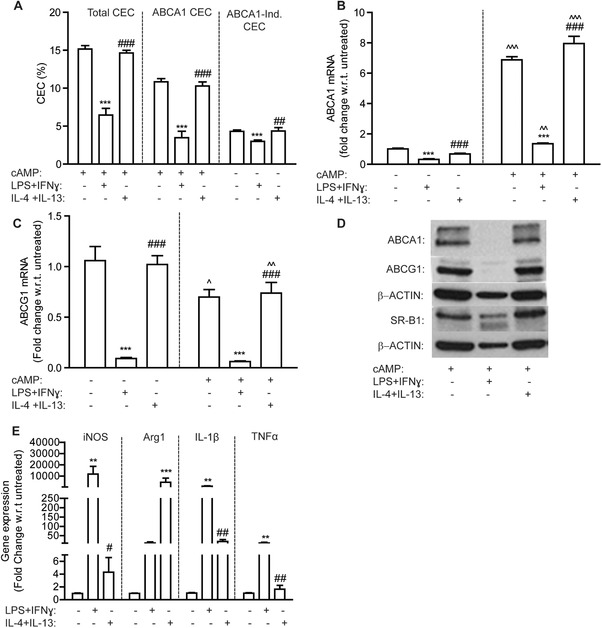
M1 macrophages exhibit reduced CEC. J774 macrophages, labeled with ^3^H‐cholesterol (1 μCi mL^–1^) in RPMI media, were stimulated ± cAMP (0.3 mm) for 1 h prior to cotreatment with polarizing cytokines for a further 18 h. M1 macrophages were polarized with LPS (100 ng mL^–1^) and IFN‐γ (20 ng mL^–1^) and M2 macrophages with IL‐4 (20 ng mL^–1^) and IL‐13 (20 ng mL^–1^). M0 macrophages were untreated. A) Total, ABCA1‐dependent, and ABCA1‐independent CEC to serum are presented (***p < 0.001 w.r.t. M0 versus M1; ^##^ *p* < 0.01, ^###^ *p* < 0.001 w.r.t. M1 versu M2). B) ABCA1 and C) ABCG1 mRNA levels were determined by RT‐PCR (*p < 0.05, **p < 0.01, ***p < 0.001 w.r.t. M0 versu M1; ^###^ *p* < 0.001 w.r.t. M1 versu M2; ^^^*p* < 0.001 w.r.t. –cAMP versus +cAMP). D) Protein levels of ABCA1, ABCG1, and SR‐B1 were measured by immunoblotting. E) mRNA levels of M1 and M2 polarization markers were measured by RT‐PCR (**p < 0.01, ***p < 0.001 w.r.t. M0 versu M1; ^#^ *p*<0.05, ^##^p<0.01 w.r.t. M1 versus M2; *n* = 3 in triplicate).

In contrast to obese ATM, macrophages polarized to a classical M1 phenotype using LPS and IFN‐γ exhibited reduced total CEC, ABCA1‐dependent CEC, and, to a lesser extent, ABCA1‐independent CEC (**Figure** 2A); compared to untreated controls (M0). cAMP treatment increased ABCA1, but not ABCG1, mRNA expression as anticipated (Figure 2B and C). ABCA1 and ABCG1 mRNA levels were suppressed in M1 macrophages compared to M0 and cAMP‐induced ABCA1 mRNA was also potently suppressed in M1 macrophages (Figure 2B). ABCA1 and ABCG1 protein levels were both suppressed in M1 macrophages relative to M0 (Figure 2D and Figure S1, Supporting InformationI). No difference in CEC or ABCA1 expression was evident upon M2 polarization (Figure 2A–D; Figure S1I, Supporting Information). M1 polarization markers (i*Nos*, *Tnfα*, and *Il‐1β*) and the M2 polarization marker arginase 1 (*arg1*) were upregulated in M1 and M2 macrophages, respectively, indicating successful polarization (Figure 2E).

### 3.3 ABCA1‐Dependent CEC Rescued in M1 Macrophages by Inhibition of the JAK/STAT Pathway

**Figure 3:**
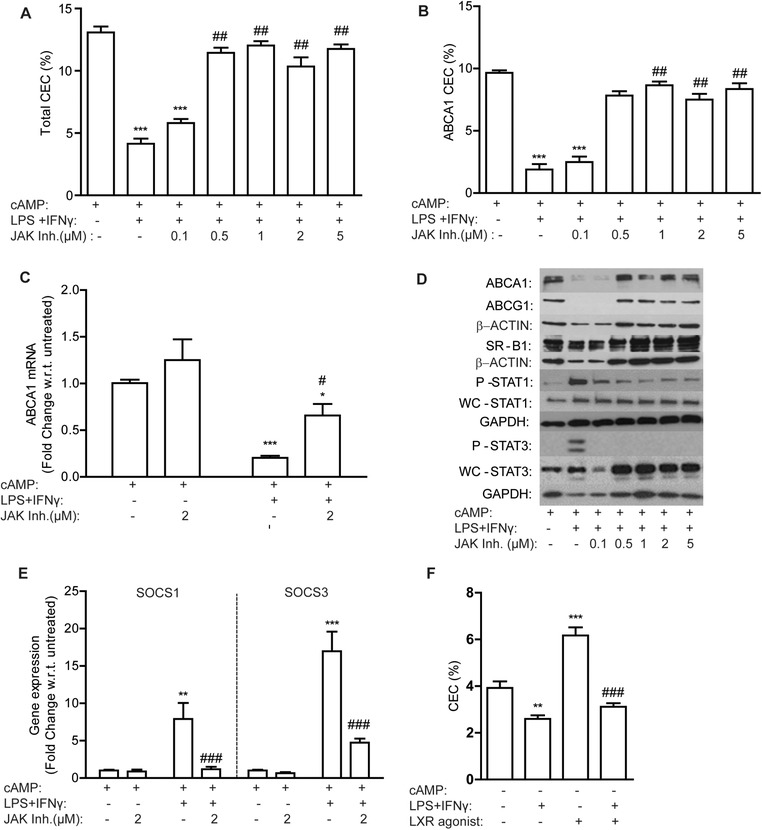
JAK/STAT pathway inhibition prevents suppression of ABCA1‐CEC in M1 macrophages. J774 macrophages, labeled with ^3^H‐cholesterol (1 μCi mL^–1^) in RPMI media were treated with increasing concentrations of a JAK/STAT pathway inhibitor ± cAMP (0.3 mm) for 2 h prior to addition of LPS (10 ng mL^–1^) and IFN‐γ (2 ng mL^–1^) for a further 18 h. A) Total CEC and B) ABCA1‐CEC to serum over 4 h is presented (***p < 0.001 w.r.t. M0 (–LPS/IFN‐γ); ^##^ *p*<0.01 w.r.t. +LPS/IFN‐γ). C) ABCA1 mRNA and D) protein expression of ABCA1, ABCG1, SR‐B1, phosphorylated (P)‐STAT1, whole‐cell (WC)‐STAT1, P‐STAT3, and WC‐STAT3 was determined. E) SOCS1 and SOCS3 mRNA levels were determined by RT‐PCR (*p < 0.05, **p < 0.01, ***p < 0.001 w.r.t. M0 (–LPS/IFNγ and –JAK inhibitor); ^#^ *p* < 0.05, ^###^ *p* < 0.001 w.r.t. +LPS/IFNγ, –JAK inhibitor). ^3^H‐cholesterol‐labeled J774 macrophages were treated ± LXR agonist (10 μm) for 2 h prior to cotreatment with M1 polarizing cytokines. F) Percentage efflux to serum was determined (**p < 0.01, ***p < 0.001 w.r.t. untreated; ^###^ *p *< 0.001 w.r.t. +LXR agonist alone; *n* = 3 in triplicate).

LPS and IFN‐γ reduced total and ABCA1‐dependent CEC (Figure S2I, Supporting Information) and induced M1 mRNA markers (*iNos* and *Il‐1β*) (Figure S2II, Supporting Information), in a dose‐dependent manner. IFN‐γ activates the JAK/STAT pathway (STAT1 and STAT3 activation) while LPS induces a type 1 interferon response, which in turn activates the JAK/STAT pathway.^9^, ^10^ We therefore hypothesized that inhibition of the JAK/STAT pathway may preserve CEC in M1 macrophages. We demonstrate that pretreatment with a pan‐JAK/STAT inhibitor could prevent loss of total‐CEC (**Figure** 3A) and ABCA1‐CEC (Figure 3B) in M1 macrophages in a dose‐dependent manner, coincident with preservation of ABCA1 mRNA and protein levels (Figure 3C and D; Figure S3I, Supporting Information). Phosphorylation of both STAT1 and STAT3 (Figure 3D;Figure S3II, Supporting Information) was prevented by JAK/STAT pathway inhibition; with corresponding reduction of their respective target genes *Socs1* and *Socs3* (Figure 3E). Furthermore, expression of the M1 marker i*Nos* was significantly reduced in cells pretreated with the JAK/STAT pathway inhibitor (Figure S2III, Supporting Information), indicative of reduced M1 polarization. More specific activators of STAT3 (IL‐10, IL‐6, and leptin) and NF‐κB (IL‐1β and TNF‐α) had no effect on CEC to serum (Figure 2IV, Supporting Information). LXR‐induced CEC to serum was also suppressed in M1 macrophages (Figure 3F).

### 3.4 Metabolic Activation of Macrophages using a Cocktail of Glucose, Insulin and Palmitic Acid Increases ABCA1‐Mediated Efflux

**Figure 4:**
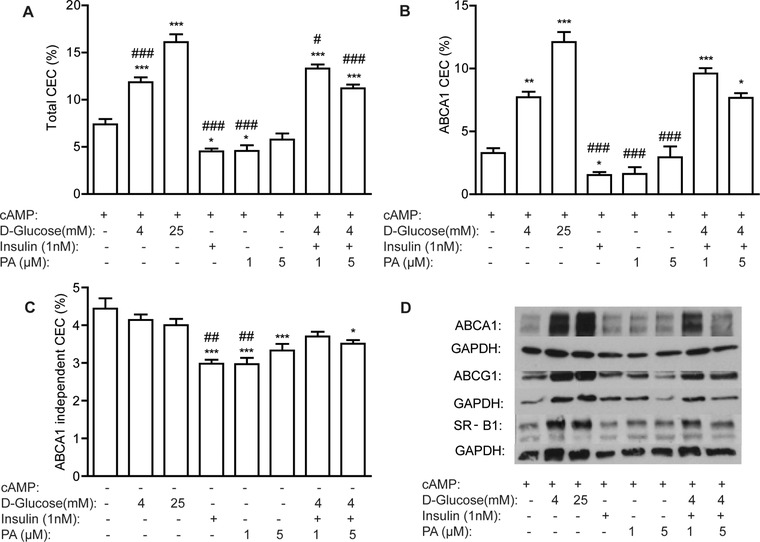
Metabolic activation enhances cAMP‐induced ABCA1 efflux in J774 macrophages. J774 macrophages, labeled with ^3^H‐cholesterol (1μCi mL^–1^) in RPMI media for 24 h were stimulated ± cAMP (0.3 mm) for 1 h and cotreated with glucose, insulin, or palmitic acid (PA) alone or in combination for 18 h in d‐glucose free DMEM. A) Total, B) ABCA1‐dependent, and C) ABCA1‐independent CEC were determined in serum over 4 h (*p < 0.05, **p < 0.01, ***p < 0.001 w.r.t. untreated control (‐glucose, ‐PA, and ‐insulin); ^#^ *p* < 0.05 and ^###^ *p* < 0.001 w.r.t. 25 mm glucose). D) ABCA1, ABCG1, and SR‐B1 protein levels were determined by immunoblotting (*n* = 3 in triplicate).

We next investigated whether metabolic stressors that are elevated in the obese microenvironment (glucose, insulin and palmitic acid) may account for changes in cholesterol trafficking within obese‐ATM. Glucose dose‐dependently increased total‐CEC and ABCA1‐CEC in J774 macrophages with no effect on ABCA1‐independent CEC (**Figure** 4A–C). cAMP‐induced ABCA1 protein expression was significantly increased in response to glucose (Figure 4D and Figure S4I, Supporting Information). l‐Glucose, an enantiomer of d‐glucose, was used as an osmotic control and no increase in total‐CEC or ABCA1 protein expression was observed with l‐glucose (Figure S5I–III, Supporting Information). Individual treatment with insulin (1 nm) or palmitic acid (1 μm) reduced total‐CEC, ABCA1‐CEC, and ABCA1‐independent CEC, while higher doses of palmitic acid (5 μm) had negligible effects (Figure 4A–C). Upon cotreatment of macrophages with glucose, insulin, and palmitic acid the glucose effect prevailed, with increased total and ABCA1‐CEC and increased ABCA1 protein expression (Figure 4A, B, and D). The effect of glucose on LXR‐driven efflux was also evaluated and by contrast, a trend toward reduced efflux was observed to both ApoA1 and serum after incubation in high‐glucose compared to normal‐glucose conditions (Figure S5IV and V, Supporting Information).

### 3.5 Enhancement of cAMP‐Induced Efflux by High Glucose is Attenuated Using a cAMP‐Response Element Binding Protein (CREB)‐Inhibitor

**Figure 5:**
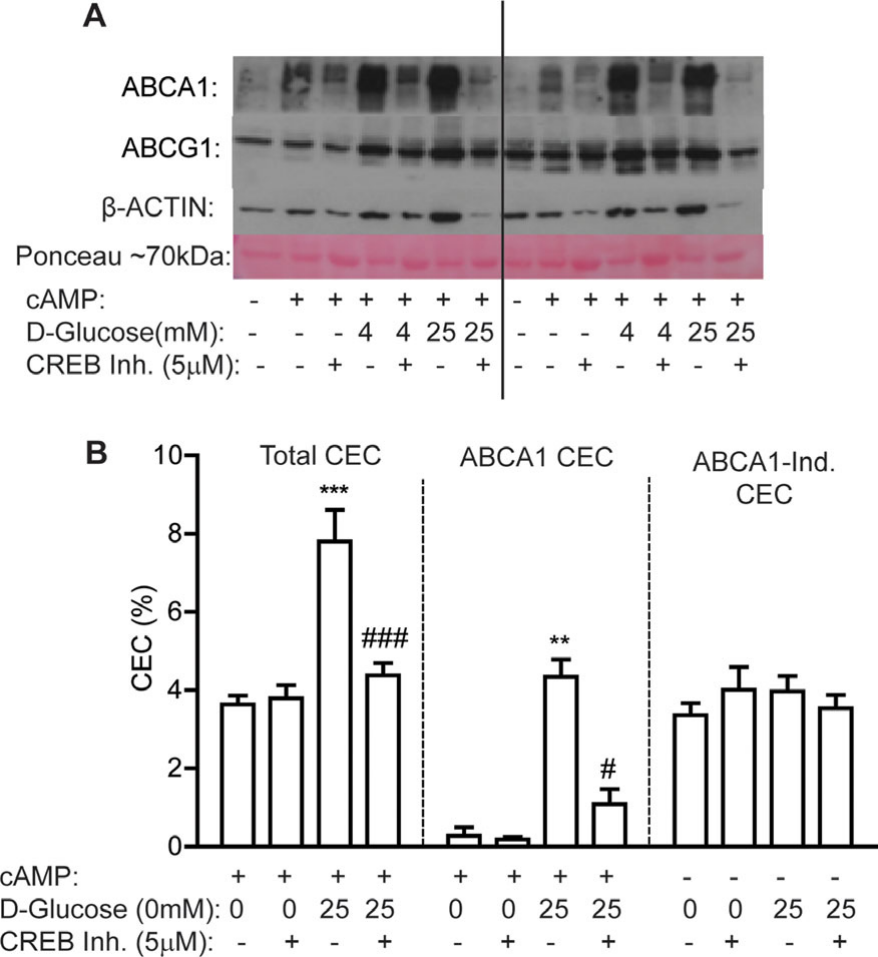
Synergy between glucose and cAMP on ABCA1 efflux is abrogated with a CREB inhibitor. J774 macrophages were treated with a CREB inhibitor (5 μm) and ^3^H‐cholesterol (1 μCi mL^–1^) in 1% RPMI media for 24 h, then cotreated with CREB inhibitor (5 μm) ± cAMP (0.3 mm) ± d‐glucose (4 or 25 mm) in d‐glucose‐free media containing 0.2% BSA for 18 h. A) ABCA1 protein levels were determined by immunoblotting. B) Total, ABCA1, and ABCA1‐independent CECs were determined in serum over 4 h (**p < 0.01, ***p < 0.001 w.r.t. untreated (‐glucose and –CREB inhibitor); ^#^ *p* < 0.05, ^###^ *p* < 0.001 w.r.t. +25 mm d‐glucose and +cAMP). (*n* = 3 in triplicate).

In the absence of cAMP, high glucose (25 mm) had no effect on CEC to serum (Figure 4C) prompting us to hypothesise that cAMP‐induced ABCA1 via CREB is the mechanism underlying enhanced ABCA1 expression and CEC. Further to this, pretreatment with a CREB inhibitor prevented cAMP‐induced ABCA1 protein expression in J774 macrophages (**Figure** 5A) and abrogated increased total and ABCA1‐CEC (Figure 5B) in response to 25 mm glucose.

## 4 Discussion

In this study, we demonstrated that microenvironmental cues in vivo have important consequences for macrophage CEC. We demonstrate that obese‐F4/80^+^ ATM, that are enriched for M1 macrophages,^1^ exhibit enhanced CEC and increased ABCA1 protein expression compared to lean‐F4/80^+^ ATM. By contrast, classical M1 activation in vitro suppresses CEC coincident with reduced ABCA1 and ABCG1 expression, effects that were mediated via activation of the JAK/STAT pathway. Metabolic activation of macrophages in vitro with glucose, insulin, and palmitic acid, could recapitulate the in vivo findings and indicate that the metabolic cues received by obese ATM may be important determinants of cellular CEC.

Chronic inflammation is a classic hallmark of obesity and in turn inflammation is intricately linked with lipid metabolism.^11^, ^12^ Yvan‐Charvet et al. demonstrated that lack of ABCA1 and ABCG1 on macrophages augments the inflammatory response with enhanced expression of TLR4 on the cell surface due to increased retention of membrane cholesterol.^5^ Furthermore, Zhu and colleagues have previously shown that myeloid deficiency of ABCA1 protects mice against *Listeria monocytogenes* infection.^13^ It is therefore plausible that under a classically inflamed environment, downregulation of ABCA1 could be beneficial to help propagate the inflammatory response. Castrillo et al. previously demonstrated that activation of TLR3 and TLR4 by microbial ligands in peritoneal macrophages blocks LXR‐induced ABCA1, and subsequent CEC.^14^ Wang et al. demonstrated a rescue of CEC after IFN‐γ treatment in Stat1^−/−^ macrophages compared to wild‐type macrophages.^15^ We similarly demonstrate a rescue of ABCA1‐mediated efflux from M1 macrophages using a JAK/STAT pathway inhibitor. In contrast to findings in murine cells, human monocyte‐derived macrophages, polarized to a M1 phenotype in vitro, exhibited a trend toward increased CEC under basal conditions.^16^ These findings demonstrate the remarkable heterogeneity that exists in response to ‘M1’ polarizing conditions in vitro and warrants caution on inference of findings derived from in vitro studies to in vivo conditions. The M1/M2 dichotomy has been increasingly challenged in recent years^17^ with greater emphasis on the importance of the tissue microenvironment in predicting macrophage fate. Indeed, it has been elegantly demonstrated that primary macrophages lose their tissue‐specific gene expression patterns after 7 days in culture indicative of the importance of microenvironmental cues in sustaining macrophage phenotype.^18^

In corroboration with these findings, Kratz et al. demonstrated that M1 macrophages derived from obese adipose tissue (metabolic cues predominate) were devoid of M1 cell surface markers (CD38, CD274, and CD319) compared to M1 macrophages derived from the airways of cystic fibrosis patients (microbial cues predominate) despite elevated *IL‐1β* and *TNF‐α* mRNA levels.^8^ Similar to our study, the authors modeled these findings in vitro, using LPS/IFN‐γ to drive a M1 phenotype and glucose, insulin and palmitate (combined) to drive a MMe phenotype and found ABCA1 to be one of the most divergently expressed proteins between these macrophage phenotypes in vitro (upregulated in MMe and downregulated in M1). The present study demonstrates that such an increase in ABCA1 is also evident in primary ATMs derived from an obese microenvironment compared to a lean microenvironment and this elevation in ABCA1 translates to increased CEC. Elucidating the specific metabolic stressor/s that are mediating this effect in vivo is extremely difficult given the complex microenvironment of adipose tissue. We took a reductionist approach and assessed three stressors that are elevated in obesity (glucose, insulin, and palmitic acid) that may be mediating this effect and determined their effects on macrophage CEC alone and in combination at physiologically relevant concentrations. However, we acknowledge that there are a multitude of other metabolites that may be contributing to these effects and the results observed are not proof of causation in vivo.

ABCA1 expression in murine macrophages is regulated by both LXRα^19^ and CREB^20^ transcription factors in a ligand specific manner. cAMP induces ABCA1 via CREB,^20^ and CREB activity has been shown to be enhanced under hyperglycaemic conditions in islet cells previously.^21^ We demonstrate that glucose dose‐dependently increased cAMP‐driven ABCA1 efflux and ABCA1 protein levels in J774 macrophages, via activation of CREB. By contrast, low‐dose palmitic acid and insulin decreased CEC. Kratz et al. previously found that palmitic acid was accountable for increased ABCA1 expression in human Mme polarized in vitro via activation of PPARγ.^8^ Fatty acids are weak inducers of PPARγ^22^ and lack of induction of ABCA1 with palmitic acid in our model was likely attributable to the lower dose used (1–5 μm) compared with Kratz et al. (400 μm). It is important to note that the cAMP‐response element in the murine ABCA1 gene is not evident in the human ABCA1 gene^20^ and this may account for different underlying mechanisms accounting for increased ABCA1 between our study and that of Kratz and colleagues. That withstanding increased ABCA1 expression is evident in obese ATM from both mice and humans^8^—whether similar mechanisms account for these effects requires investigation.

In contrast to cAMP‐induced CEC, LXR‐induced CEC was suppressed in J774 macrophages under hyperglycemic conditions. This was consistent with findings by Hussein et al. who demonstrated that d‐glucose (25 mm) suppressed LXR‐dependent expression of ABCA1.^23^ Patel et al. previously demonstrated reduced ABCA1 protein expression in circulating leukocytes from patients with type 2 diabetes.^24^ Tang et al. similarly showed peritoneal macrophages from diabetic mice had reduced ABCA1 and CEC.^25^ Therefore high‐glucose can have divergent effects on ABCA1‐expression dependent on the predominant transcription factor present within the cell. The ability of HDL to support cholesterol efflux is also modulated in individuals with the metabolic syndrome, particularly via SR‐BI and ABCG1 pathways, indicative that a multitude of steps in reverse cholesterol transport are reduced in the setting of cardiometabolic disease.^26^ Our study has shown that the microenvironment of obese adipose tissue results in increased ABCA1 activity, in contrast to peripheral cells from diabetic patients, again indicative that the primary source of tissue macrophages is a critical determinant of cellular ABCA1.

There are several limitations to the current study, most notably the use of in vitro models to mimic the in vivo environment. Indeed, our findings warrant caution about the over‐interpretation of the biological significance of findings derived from cells polarized in vitro. While physiological concentrations of metabolic stressors were used in our study we cannot rule out a role for other metabolites in vivo including modified lipids, oxysterols, cholesterol, and lipid derivatives such as ceramides. Furthermore, the hypoxic conditions within obese adipose tissue and the heterogenic cell–cell communication that occurs in vivo is not replicated in vitro. Another limitation is the use of murine macrophages which exhibit differential regulation of ABCA1 compared to human macrophages. It will therefore be critical to validate our findings in human ATM in the future. It will also be of great interest to establish the impact of obesity on cholesterol influx into ATM to harness greater understanding about bidirectional cholesterol trafficking within the obese microenvironment.

These novel findings demonstrate remarkable plasticity of macrophages to respond to their local environment with specific modulation of ABCA1 depending on whether classical pro‐inflammatory or metabolic cues predominate. Enhanced ABCA1‐CEC of metabolic macrophages, despite a pro‐inflammatory phenotype, would be beneficial to prevent lipotoxicity by acquired lipids. By contrast, suppression of ABCA1‐CEC and enhanced TLR signaling would be beneficial in combating infection in classically inflamed M1 macrophages.^13^ Modulation of ABCA1 within the macrophage membrane is likely an important cellular adaptation for optimal function that is governed by external environmental factors.

## Conflict of Interest

The authors declare no conflict of interest.

## Supporting information

Supplementary

Supplementary

Supplementary

Supplementary

Supplementary

Supplementary

Supplementary
