# Supplementary figures and images for: Nutritionally Derived Metabolic Cues Typical of the Obese Microenvironment Increase Cholesterol Efflux Capacity of Adipose Tissue Macrophages
Source: Mol Nutr Food Res. 2018 Nov 20;63(2):1800713. doi: 10.1002/mnfr.201800713 (PMC6492173; doi:10.1002/mnfr.201800713)

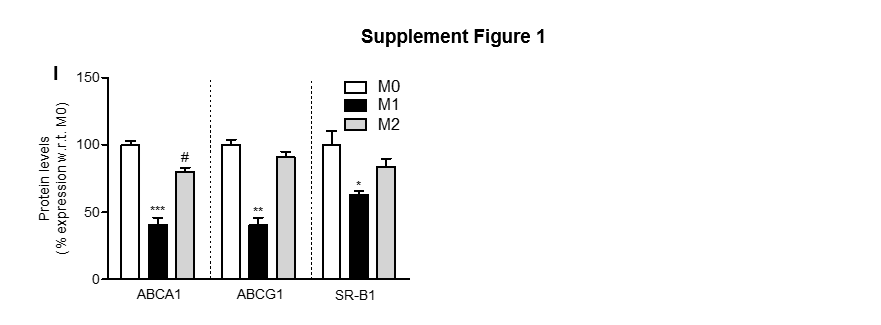

Supplement: Supplementary file 1 — Supplementary [file MNFR-63-na-s001.tif]

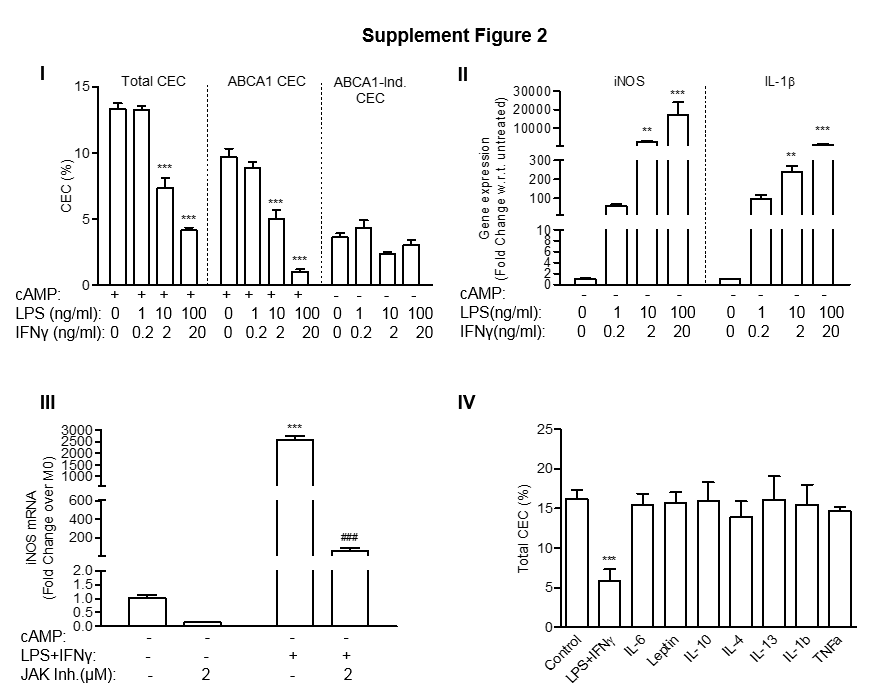

Supplement: Supplementary file 2 — Supplementary [file MNFR-63-na-s002.tif]

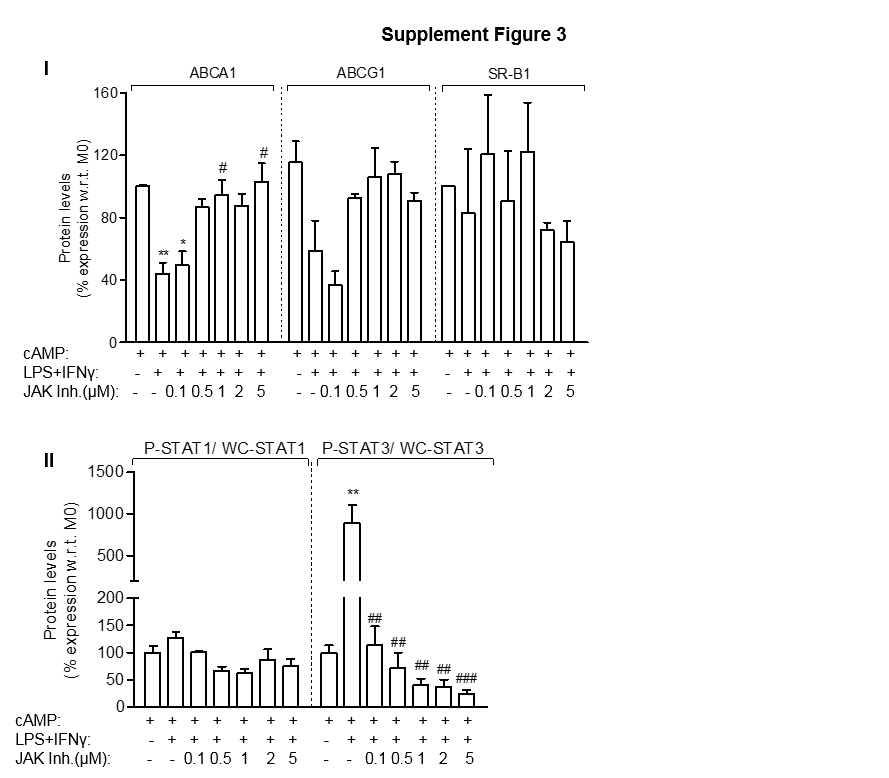

Supplement: Supplementary file 3 — Supplementary [file MNFR-63-na-s003.tif]

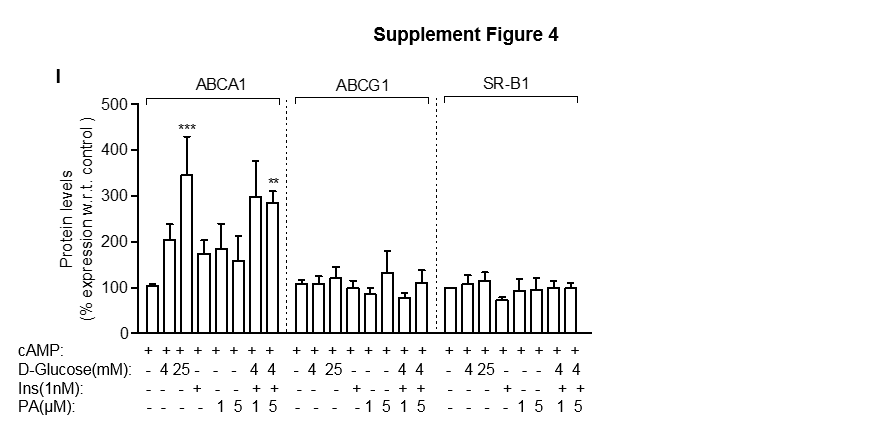

Supplement: Supplementary file 4 — Supplementary [file MNFR-63-na-s004.tif]

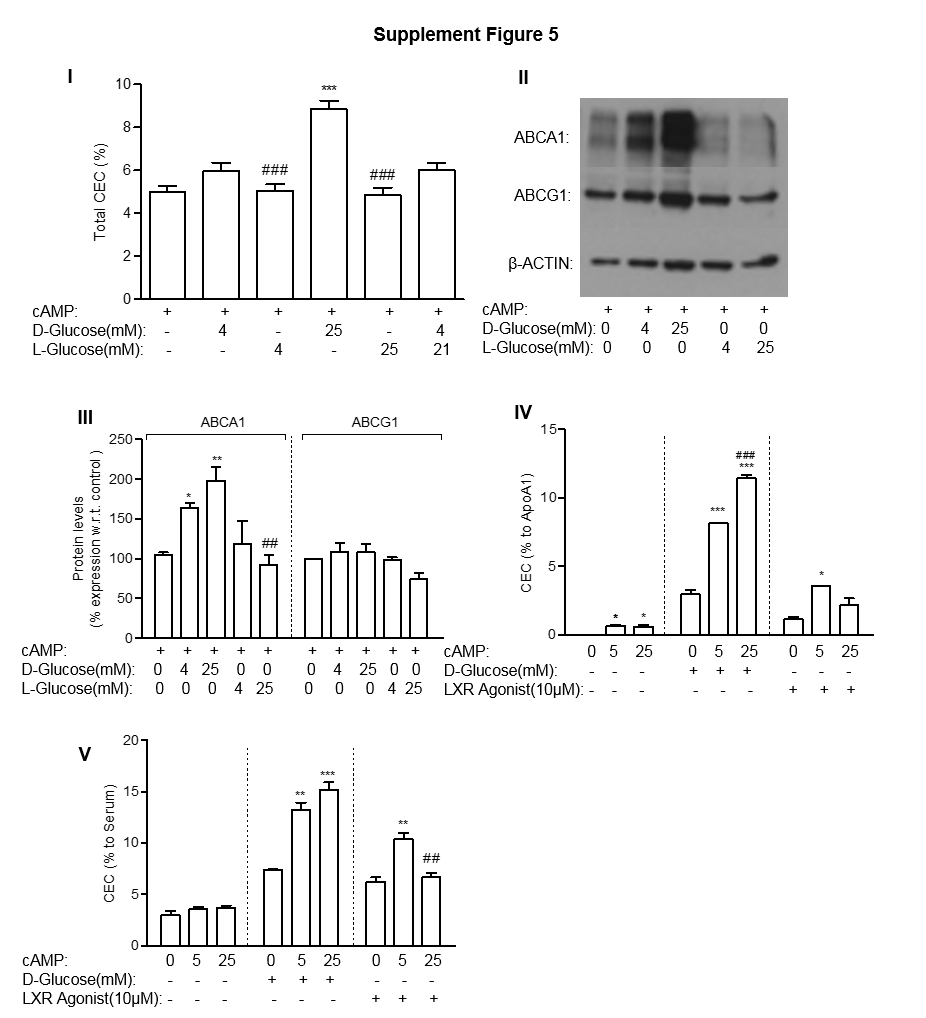

Supplement: Supplementary file 5 — Supplementary [file MNFR-63-na-s005.tif]

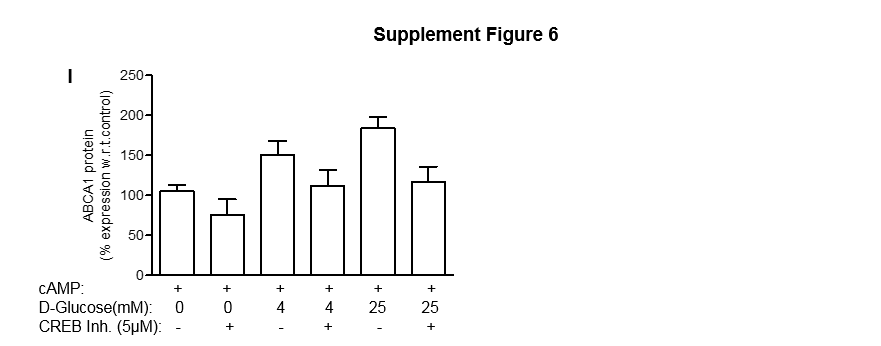

Supplement: Supplementary file 6 — Supplementary [file MNFR-63-na-s006.tif]
