# Supplementary material for: Nutritionally Derived Metabolic Cues Typical of the Obese Microenvironment Increase Cholesterol Efflux Capacity of Adipose Tissue Macrophages
Source: Mol Nutr Food Res. 2018 Nov 20;63(2):1800713. doi: 10.1002/mnfr.201800713 (PMC6492173; doi:10.1002/mnfr.201800713)
Supplement: Supplementary file 7 — Supplementary [file MNFR-63-na-s007.doc]

**Supplement Section**

**Supplement Figure Legends**

**Supplement Figure 1. (I) Densitometry for Figure 1C.** ABCA1, ABCG1, SR-B1 protein expression was determined by immunoblot and densitometry analyzed by Image J software. Densitometry data was normalized to β-actin and represented as a percentage of M0 (*p<0.05, **p<0.01, ***p<0.001 w.r.t. M0, ^#^p<0.05 w.r.t. M1, n=3).

**Supplement Figure 2. Effects of inflammation on macrophage CEC and M1 polarization markers.** J774 macrophages, labeled with ^3^H-cholesterol (1µCi/ml) in RPMI media, were stimulated ± cAMP (0.3mM) for 1h and then co-treated ± cAMP ± increasing doses of LPS and IFNγ for 18h. (I) Total, ABCA1-dependent and ABCA1-independent CEC to serum over 4h is presented (***p<0.001, w.r.t. control (+cAMP, -LPS/IFNγ, n=3). (II) J774 macrophages were treated with increasing concentrations of LPS and IFNγ for 18h and mRNA levels of *iNOS* and *IL-1β* were measured by RT-PCR (**p<0.01, ***p<0.001 w.r.t. untreated, n=3). (III) *iNOS* mRNA was determined in M1 (LPS+IFNγ) macrophages pre-treated ± 2µM JAK/STAT pathway inhibitor (**p<0.01, ***p<0.001 w.r.t. untreated; ^##^p<0.05, ^###^p<0.001 w.r.t. LPS+IFNγ, n=3). (IV) J774 macrophages, labeled with ^3^H-cholesterol (1µCi/ml), were stimulated + cAMP (0.3mM) for 1h then co-treated with LPS (100ng/ml) +IFNγ (20ng/ml), IL-6, Leptin, IL-10, IL-4, IL-13, TNFα (20ng/ml) or IL-1β (10ng/ml) for 18h. Total CEC to serum was determined (***p<0.001 w.r.t. untreated control, n=3).

**Supplement Figure 3.** **Densitometry for Figure 2D.** J774 macrophages were co-treated with increasing concentrations of a JAK/STAT pathway inhibitor + cAMP (0.3mM) for 2h prior to co-treatment with LPS (10ng/ml) &IFNγ (2ng/ml) for a further 18h. (I) ABCA1, ABCG1 and SR-B1 protein expression was determined by immunoblot and densitometry analyzed by Image J software. Densitometry data was normalized to β-actin and represented as percentage of M0. (II) Phosphorylated STAT1/ Whole Cell STAT1 and Phosphorylated STAT3/ Whole Cell STAT3 normalized to GAPDH and represented as percentage expression of M0 (*p<0.05,**p<0.01 w.r.t. M0 (+cAMP, -LPS/IFNγ and -JAK inhibitor); ^#^p<0.05 ^##^p<0.01, ^###^p<0.001 w.r.t. M1 (+cAMP, +LPS+IFNγ and -JAK inhibitor), n=3).

**Supplement Figure 4. (I) Densitometry for Figure 4D.** J774 macrophages were stimulated + cAMP (0.3mM) for 1h and co-treated with glucose, insulin or palmitic acid alone or in combination for 18h. ABCA1, ABCG1 and SR-B1 protein expression was determined by immunoblot and densitometry analyzed by Image J software. Densitometry data was normalized to GAPDH (**p<0.01, ***p<0.001, w.r.t. +cAMP, n=3).

**Supplement Figure 5. Modulation of CEC in metabolic macrophages.** (I&II) J774 macrophages, labeled with ^3^H-cholesterol (1µCi/ml), were stimulated + cAMP (0.3mM) for 1h and co-treated with D-glucose (4, 25mM) or L-glucose (4, 21 or 25mM) for 18h. (I) Total CEC to serum (***p<0.001 w.r.t. +cAMP, -glucose; ^###^p<0.001 w.r.t. 25mM D-Glucose, n=3), (II) cellular ABCA1 protein expression and (III) corresponding densitometry normalized to β-actin presented (*p<0.05, **p<0.01 w.r.t. +cAMP, -glucose; ^##^p<0.01 w.r.t. 25mM glucose, n=3).  (IV&V) ^3^H-cholesterol labeled J774 macrophages were treated ± cAMP (0.3mM) ± LXR agonist (10µM) ± D-glucose (5 and 25mM) for 18h and cholesterol efflux to (IV) ApoA1 and (V) serum determined (*p<0.05 w.r.t.0mM D-glucose; ^###^p<0.001 w.r.t 5mM D-glucose, n=3).

**Supplement Figure 6. (I) Densitometry for Figure 5A.** J774 macrophages were treated with CREB inhibitor (5µM) for 24h and then co-treated with CREB inhibitor (5µM) + cAMP (0.3mM), ± D-Glucose (4 or 25mM) in D-glucose free media containing 0.2% BSA for 18h. ABCA1 protein expression was determined by immunoblot and densitometry analyzed by Image J software. Densitometry data was normalized to ponceau (n=3).
